# Winter Refuge for *Aedes aegypti* and *Ae. albopictus* Mosquitoes in Hanoi during Winter

**DOI:** 10.1371/journal.pone.0095606

**Published:** 2014-04-21

**Authors:** Takashi Tsunoda, Tran Chi Cuong, Tran Duc Dong, Nguyen Thi Yen, Nguyen Hoang Le, Tran Vu Phong, Noboru Minakawa

**Affiliations:** 1 Department of Vector Ecology and Environment, Institute of Tropical Medicine (NEKKEN), Nagasaki University, Nagasaki, Japan; 2 Department of Medical Entomology and Zoology, National Institute of Hygiene and Epidemiology, Hanoi, Vietnam; Kansas State University, United States of America

## Abstract

Dengue occurs throughout the year in Hanoi, Vietnam, despite winter low temperatures <10°C. During July 2010 to March 2012, we surveyed monthly for *Aedes* larvae and pupae in 120 houses in 8 Hanoi districts. *Aedes albopictus* preferred discarded containers in summer and pupal density drastically decreased in winter. *Aedes aegypti* preferred concrete tanks and this preference increased in winter. Even in winter, the lowest water temperature found in concrete tanks was >14°C, exceeding the developmental zero point of *Ae. aegypti*. Although jars, drums and concrete tanks were the dominant containers previously (1994–97) in Hanoi, currently the percentage of residences with concrete tanks was still high while jars and drums were quite low. Our study showed that concrete tanks with broken lids allowing mosquitoes access were important winter refuge for *Ae. aegypti*. We also indicate a concern about concrete tanks serving as foci for *Ae. aegypti* to expand their distribution in cooler regions.

## Introduction

Dengue fever and dengue haemorrhagic fever are very important viral diseases, with an estimated 50 million infections every year and around 3.6 billion people living in areas at risk [1]. Dengue virus is transmitted by *Aedes aegypti* (L.) and in Southeast Asia to a lesser extent also by *Aedes albopictus* (Skuse) (Diptera: Culicidae) [2]. In Hanoi northern Vietnam, there were two major outbreaks in 1998 and 2009 and dengue incidence has been increasing yearly since 1999 [3]. Transmission peaks in late summer; however, many dengue cases occur in Hanoi every winter [4], [5].

Although Hanoi has a sub-tropical climate, winters are cool, with minimum temperatures around 10°C [6]. Both *Ae. aegypti* and *Ae. albopictus* are found in Hanoi [3]. In Hanoi, these species may experience seasonal constraints in activity due to cold temperature, as the developmental zero point of *Ae. aegypti* is 13.3°C [7] and that of *Ae. albopictus* is 12.0°C [8].

Mosquitoes in temperate zones have efficient overwintering mechanisms and hibernation in the egg stage is practiced by most *Aedes* species [9]. In short day-length conditions, *Ae. albopictus* collected from East Asia (Shanghai, Beijing, Japan and Korea) enters into diapause [10]. Though *Ae. albopictus* from Southeast Asia (Taipei, Hong Kong, Thailand, and Malaysia) does not enter into diapause in short day conditions [10], little is known about overwintering of *Ae. albopictus* in Vietnam.

Within the urban environment in many developing countries, densely packed housing and inadequate drinking-water supplies, poor garbage collection services, and surface-water drainage systems combine to create favorable habitats for the proliferation of insect vectors and reservoirs of communicable diseases [11]. In Hanoi, urbanization has been proceeding rapidly and infrastructure has been developing. During a 1994–1997 survey in Hanoi, the major habitat for *Aedes* immatures were ceramic jars, drums, and concrete tanks [12]. However, in the 15 years since this survey, the situation may have changed, and urbanization may change *Aedes* habitat and behavior.

Overall in southeast China, the winter minimum and maximum temperatures have been increasing as has diurnal temperature range. Where rapid urbanization has occurred, land-use changes from urbanization have created urban heat islands [13]. Vietnam has also experienced dramatic economic growth since the Doi Moi Policy started in 1986, also contributing to rapid urbanization in Hanoi. In Hanoi, warming via urbanization may result in greater mosquito survival during winter. However, little is known about winter activity of dengue vectors, as there is little transmission then. Our purpose was to investigate the seasonal activity of *Ae. aegypti* and *Ae.albopictus* in Hanoi, especially in winter.

## Materials and Methods

### Study Area

The study area included eight districts (Hoan Kiem, Dong Da, Hai Ba Trung, Hoang Mai, Thanh Xuan, Thanh Tri, Tu Liem, and Ha Dong) in Hanoi City, Vietnam. These districts had many dengue cases in 2008 and 2009 [4], [5]. We selected 15 houses randomly in each district, a total of 120 houses monthly.

### Entomological Survey

All indoor and outdoor containers were surveyed to confirm the presence of *Aedes* immatures. Pupae were brought to the laboratory and reared to adults for species identification. Buckets, drums, jars, concrete tanks, toilet concrete tanks, and wells were sampled by sweeping them five times with a small net. Bonsai, trees grown in containers, plant saucers, and aquarium without fish were included in others. The number of pupae was estimated from mean interval percentage recovery [14], and small containers were emptied to collect pupae. All water-holding containers, both indoors and outdoors, were inspected only after obtaining the necessary permission from the occupants.

### Temperature Measurement

**Figure 1:**
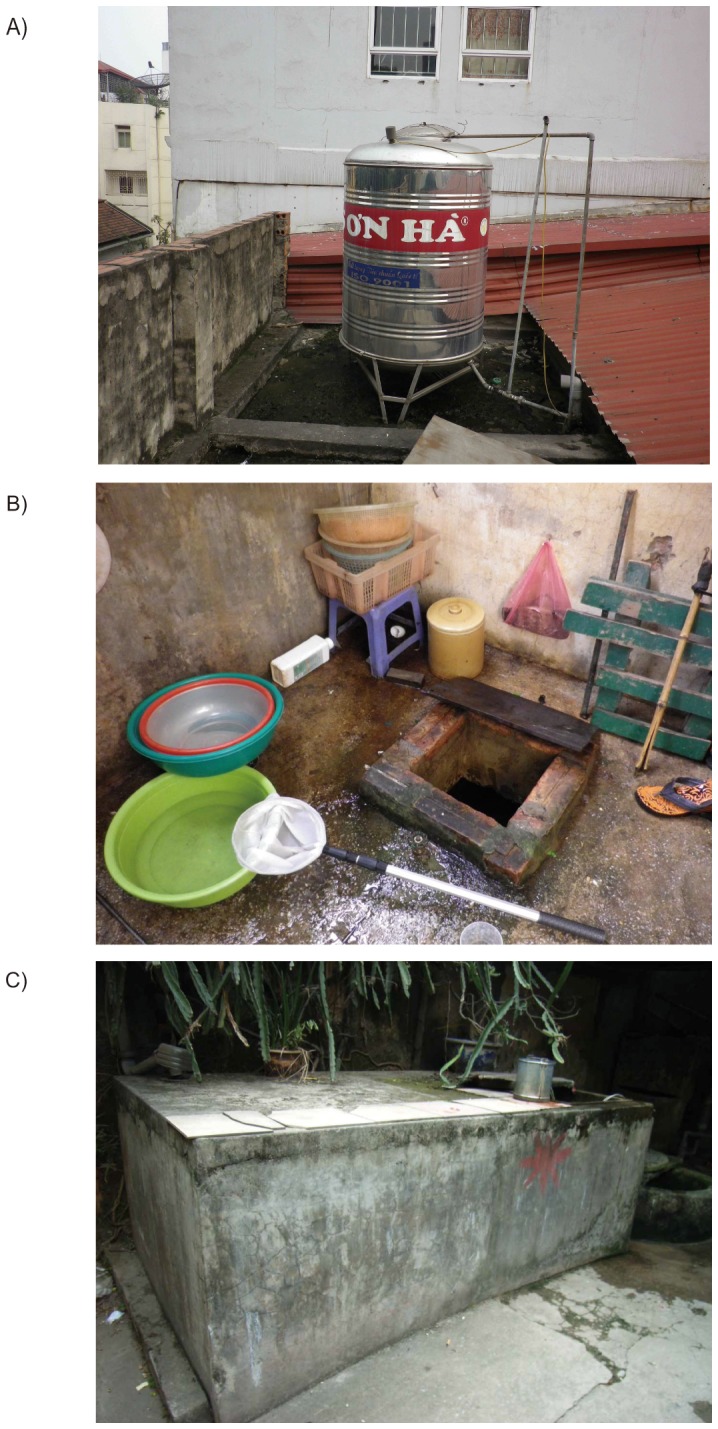
Typical water tanks in Hanoi. A) Roof-top metal tank. B) Underground concrete tank. C) Above-ground concrete tank.

Our preliminary survey showed that residents seldom use ceramic jars and drums for storing water and concrete tanks are preferred. January is the coldest month in Hanoi [6]. We measured water temperatures in seven concrete tanks and one metal tank by data loggers (HOBO Pendant Temperature/Light Data Logger 8K-UA-002-08, Onset, MA, USA) in January 2011 and January 2012 (Fig. 1). The concrete tanks in Hoang Mai in 2012 and Tu Liem in 2011 and 2012 were outdoors and set in the ground. The other concrete tanks were in basements (Dong Da, Hai Ba Trung, Hoang Mai in 2011, Thanh Xuan, Thanh Tri, and Ha Dong). A metal tank in Hoan Kiem was under the roof of a house in 2011 and on the roof of another in 2012. We also measured room temperature in health centers of Hoang Mai in January 2011 and 2012 and Ha Dong in January 2011 using the same data logger. The room temperature of Ha Dong in January 2012 was measured by a thermo recorder (TR-72U, T&D Corporation, Matsumoto City, Japan).

We also obtained the outside air temperature data measured in Dong Da district from January 2010 to October 2012 [15].

### Statistical Analysis

The lowest temperature and January mean temperature data in 2011 and 2012 were subjected to analysis of variance (ANOVA) to compare the values of different medium and location. When the F-test was significant (p<0.05), the treatment means were compared using Tukey’s significantly different test. To examine which type of container was used by mosquitoes in summer and winter, habitat preference was analyzed by G-test. Similar types of containers were grouped if expected frequencies were <3. Multiple correspondence analysis was conducted to analyze the relationship between species (*Ae. aegypti* and *Ae. albopictus*), season (summer and winter) and container type. Mutiple correspondence analysis is a graphic technique that shows each category as a point in a type of scatter plot. The positions of the category-points on the graph mean similarity or association between categories. All data were analyzed using R (version 2.6.2) software.

## Results

The lowest Hanoi outside air temperature in January was 9.0°C in 2011 and 8.0°C in 2012 [15]. The mean outside air temperatures were lower than room temperatures, metal tank, and the concrete tank temperatures (Table 1). The mean concrete tank water temperature was higher than other temperatures in 2011 and 2012. The difference was not significant between the mean of outside air temperature and metal tank water temperature. However, outside air temperature and room temperature were significantly different. The mean outside air temperature was lowest in January 2011 and 2012, though it was not significantly different from means of the room temperature and metal tank water temperature (Table 2). Concrete tanks were warmer in January 2011 and 2012 compared to outside and room temperatures.

**Table 1:** The lowest temperature in 2011 and 2012.

Medium	Place	n	Temperature (°C) (mean±SD)5)
Air	Outside1)	2	8.5±0.7 a
	Room2)	4	13.4±1.0 b
Water	Metal tank3)	2	11.3±1.2 ab
	Concrete tank4)	14	18.2±1.9 c

1) National Hydro-Meteorological Service (2012).

2) Room temperature in public health centers of Hoang Mai and Ha Dong districts.

3) Metal tank set in Hoan Kiem district.

4) Concrete tanks set in seven districts (Dong Da, Hai Ba Trung, Hoang Mai, Thanh Xuan, Thanh Tri, Tu Liem, and Ha Dong).

5) Different letters following means in a row indicate significant difference at *p*<0.05 by Tukey’s test.

**Table 2:** January average temperature in 2011 and 2012.

Medium	Place	n	Temperature (°C) (mean±SD)5)
Air	Outside1)	2	13.7±1.3 a
	Room2)	4	16.2±1.2 a
Water	Metal tank3)	2	17.3±1.6 ab
	Concrete tank4)	14	20.0±1.7b

1) National Hydro-Meteorological Service (2012).

2) Room temperature of Public health centers in Hoang Mai and Ha Dong.

3) Metal tank set in Hoan Kiem.

4) Concrete tanks set in seven districts (Dong Da, Hai Ba Trung, Hoang Mai, Thanh Xuan, Thanh Tri, Tu Liem, and Ha Dong).

5) Different letters following means in a row indicate significant difference at *p*<0.05 by Tukey’s test.

**Figure 2:**
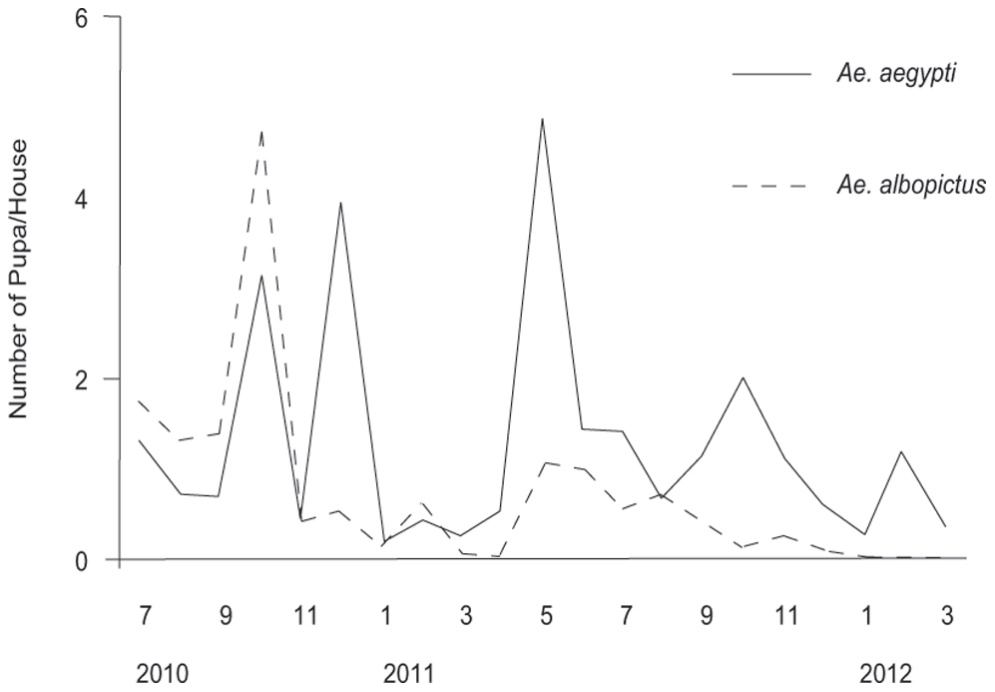
Seasonal occurrence of *Ae. aegypti* and *Ae. albopictus* in Hanoi from July 2010 to March 2012. Error bars indicate standard error.

Peaks of *Ae. aegypti* pupation were seen in October and December, 2010, and in May and October, 2011 (Fig. 2). Pupation of *Ae. albopictus* peaked only twice October 2010 and May 2011. Compared to *Ae. albopictus*, the density of *Ae. aegypti* was higher during winter.

Concrete tanks, flower vases, and discarded containers were abundant both in summer and winter (Table 3). Discarded containers were most abundant (31.4% of all habitat types) in winter, and 368 discarded bottles were examined in one house in March 2011. *Aedes aegypti* preferred concrete tanks through the year. Forty-five percent of all *Ae. aegypti* were collected from concrete tanks in summer, and 71% of all *Ae. aegypti* were from concrete tanks in winter. For *Ae. aegypti*, the frequency of preferred containers was significantly different between summer and winter (G = 292.68 d.f. = 5, *P*<0.001). Habitat preference was significantly different between *Ae. aegypti* and *Ae. albopictus* in summer (G = 654.63, d.f. = 5, *P*<0.001) and in winter (G = 81.84, d.f. = 5, *P*<0.001). Although most *Ae. albopictus* were collected from discarded containers, the frequency of preferred containers in summer was not concentrated on a particular container, as was the case for concrete tanks in *Ae. aegypti*. However, in the winter, most *Ae. albopictus* were collected from concrete tanks (41%) with 38% in other containers. There was a significant difference in habitat preference between summer and winter for *Ae. albopictus* (G = 124.37, d.f. = 5, *P*<0.001).

**Table 3:** Relative percentages of containers (number of containers), *Ae. aegypti* and *Ae. albopictus* collected from containers from July to October and from December to March in 2010 and 2011.

Container type1)	July – October			December – March		
	container	*aegypti*	*albopictus*	container	*aegypti*	*albopictus*
	(n = 2,280)	(n = 1,438)	(n = 1,302)	(n = 2,173)	(n = 992)	(n = 175)
Bucket	17.4(397)	27.6(397)	10.5(137)	9.5(206)	5.4 (53)	0 (0)
Drum	1.0 (22)	0.5 (7)	0.3 (3)	0.6 (14)	0 (0)	3.8 (7)
Flower vase	19.2(437)	2.1 (30)	3.3 (43)	21.2(460)	1.1 (11)	1.7 (3)
Jar	3.6 (82)	9.0 (130)	17.4(227)	3.3 (72)	4.0 (40)	5.7 (10)
Concrete tank	24.4(556)	44.7(643)	15.4(200)	20.8(453)	70.6(700)	40.8(71)
TCT2)	0.2 (5)	0.3 (4)	0 (0)	0.3 (6)	0 (0)	0 (0)
Tire	0.8 (19)	0.1 (1)	2.8 (37)	0.2 (4)	0 (0)	0 (0)
Discarded	18.3(417)	5.7 (82)	30.0(390)	31.4(683)	2.2 (22)	9.7 (17)
Well	1.5 (34)	0.7 (10)	1.0 (13)	1.3 (29)	0.7 (7)	0 (0)
Others3)	13.6(311)	9.4(135)	19.4(252)	11.3(246)	16.0(159)	38.3 (67)

1) Values are grouped as follows so that resulting expected frequencies are large enough; Bucket+Drum, Concrete tank+Toilet concrete tank, Tire+Discarded.

2) Toilet concrete tank.

3) Others include Bonsai, plant saucer, and aquarium.

**Figure 3:**
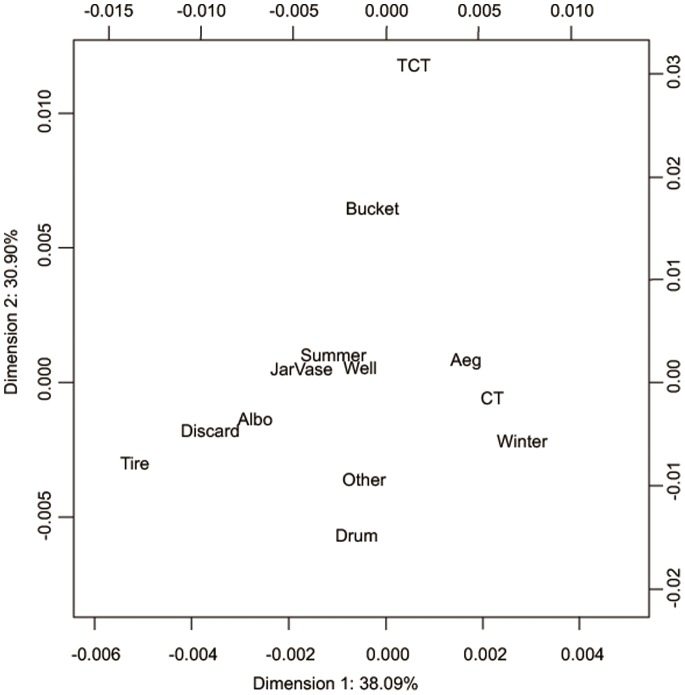
Multiple correspondence analysis of mosquito species, season, and container type. Mosquito species (*Ae. aegypti* and *Ae. albopictus*), season (summer and winter), and container type (bucket, concrete tank, discarded container, drum, jar, other containers, tire, toilet concrete tank, and flower vase). Mos. aeg; *Ae. aegypti*, Mos. albo; *Ae. albopictus*, C.; Container, CT; concrete tank, Discard; discarded container, Other; other containers, S; Summer, TCT; toilet concrete tank, W; Winter.

Multiple correspondence analysis enabled 68.99% of total inertia to be explained by two dimensions, with the first accounting for 38.09% and the second 30.90% (Fig. 3). The analysis showed that *Ae. albopictus* was closely related to discarded containers. Meanwhile, concrete tanks were related to both *Ae. aegypti* and winter.

## Discussion

Cold temperatures may act as a strong ecological constraint over possible range expansions of *Ae. aegypti* and *Ae. albopictus* [16]. Certain populations of *Ae. albopictus* may extend their summer range considerably north of the 10°C cold month isotherms, but they are not able to survive during cold winter months, which prevents the establishment of permanent populations [9]. Climatic variability and extreme temperatures will have more important effects on reproductive success and population dynamics than winter warming [17].

It may be presumed that *Ae. aegypti* and *Ae. albopictus* use different strategies to survive in winter in Hanoi. *Aedes aegypti* does not enter into diapause [18]. Our results show that *Ae. aegypti* continued to develop throughout the winter in containers that kept water warm, as the development zero point of *Ae. aegypti* is 12–13°C [7], [19], [20]. However, it is hard for *Ae. aegypti* adults to fly and search for a food in winter, because they cannot fly below 17°C [18]. Winter can impose a severe physiological stress on insects, and overwintering sites that are above the ground are generally colder and experience more variable temperature regimes than those underground [21]. Winter severity varies both temporally and spatially and many insects are adapted to take advantage of the milder conditions that can occur [21]. In our study, the average temperature of a room in January was below 17°C in Hanoi, with outside temperatures much lower. As a consequence, the time and space for *Ae. aegypti* adults to fly and feed might be limited in winter.

*Aedes albopictus* originating from Taipei, Hong Kong, and Thailand do not enter into diapause [10], which implies that *Ae. albopictus* from Hanoi may also be a non-diapause race. However, there were fewer *Ae. albopictus* in winter in Hanoi, compared with *Ae. aegypti*. Eggs of *Ae. albopictus* collected from Hanoi delayed hatching under short day length conditions [Tsunoda *et al*., unpublished data]. These results suggest that most *Ae. albopictus* enter into diapause and others continue to grow, an apparent bed-hedging strategy [22].

Migration may be an alternative strategy to escape from severe winters. In the Shanghai area of China, diapausing adults of *Culex tritaeniorhynchus* are blown southward for at least 35 km, possibly as far as 200 km, in autumn [23]. The maximum flight distance of *Ae. aegypti* is usually 100–200 m [24]–[27], although, in some particular situations, this species can fly distances >400–600 m [28], [29]. The maximum flight distance of *Ae. albopictus* is longer than that of *Ae. aegypti*, but at most 100–1,000 m [30], [31]. Therefore, both *Ae. aegypti* and *Ae. albopictus* might stay near their natal habitat in winter.

Our results show that *Ae. albopictus* preferred discarded containers mostly in summer but not in winter. It is likely that water temperature in a bottle and other discarded outside containers fluctuates with outside air temperature. It seems to be difficult for *Ae. albopictus* to grow in discarded containers in winter, because the average outside temperature in January is close to its developmental zero point, 11–12°C [8], [19].

Although *Ae. aegypti* is highly endophilic, *Ae. albopictus* is exophilic in Thailand [32], which suggests for habitat segregation between the species. However, as room air was warmer than outside air in winter, it will be advantageous for *Ae. albopictus* adults to fly into and take a blood meal in a house. We should point out that the house-entering behavior of *Ae. albopictus* may be influenced by the presence of *Ae. aegypti*.

Over the last 15 years, the distribution of containers for *Aedes* mosquitoes has changed in Hanoi. The entomological survey conducted in 1994–1997 showed that concrete tanks (38.9% in total containers), jars (30.2%), and drums (26.0%) were abundant [12]. However, in our entomological survey, the relative percentages of jars (3.3–3.6%) and drums (0.6–1.0%) were low, though concrete tanks were still high (20.8–24.4%). The reason why residents do not currently use jars and drums is attributed to the increasing popularity of tap water over the last 15 years. Residents usually keep tap water in an underground or above-ground concrete tank. The water is automatically pumped up to a rooftop tank, since tap water pressure is very weak. As a result, people retain existing tanks when they remodel a home or build a new one if a new home is constructed. Since some concrete tanks may be sealed with putty or covered by plastic sheeting and are difficult to detect, our estimate of the number of tanks may be low.

Our study showed that concrete tanks are important habitat for overwinter-survival of *Ae. aegypti* in Hanoi. Here, as well as potentially in other countries, if such domestic water systems are encouraged, these concrete tanks could serve as foci for range expansion of *Ae. aegypti* in cooler regions.
